# Psychometric Evaluation of the Posttrauma Risky Behaviors Questionnaire: Item Response Theory Analyses

**DOI:** 10.1177/10731911211036760

**Published:** 2021-07-30

**Authors:** Prathiba Natesan Batley, Ateka A. Contractor, Nicole H. Weiss, Sidonia E. Compton, Matthew Price

**Affiliations:** 1 Brunel University London, London, UK; 2 University of North Texas, Denton, TX, USA; 3 University of Rhode Island, Kingston, RI, USA; 4 University of Vermont, VT, USA

**Keywords:** Posttrauma Risky Behaviors Questionnaire, psychometrics, item response theory, differential item functioning, gender

## Abstract

The Posttrauma Risky Behaviors Questionnaire (PRBQ) assesses extent of engagement in posttrauma reckless and self-destructive behaviors (RSDBs). Given PRBQ’s recent development with limited psychometric investigations, we used item response theory to examine (a) item analysis, (b) person fit, and (c) differential item functioning (DIF) across gender-based groups and two different samples. One sample included 464 participants reporting potentially traumatic experiences (Mechanical Turk [MTurk], recruited online), and the other sample included 171 trauma-exposed women reporting current intimate partner violence and substance use (recruited in-person). All PRBQ items contributed to the RSDB scale, and all PRBQ items and the PRBQ scale provided maximum information for high levels of the RSDB latent trait. Seven and 11 items were conceptualized as low information items in the MTurk and intimate partner violence samples, respectively. Eight MTurk participants’ responses did not fit the overall pattern of responses as expected. Seven items were flagged for DIF between the two samples, and eight items were flagged for DIF between men and women in the MTurk sample. However, all effect sizes were <8%. Conclusively, results suggest good psychometric properties for the PRBQ and support its use to compare RSDBs across different samples and gender-based groups.

Evidence suggests that trauma-exposed individuals are more likely to engage in reckless and self-destructive behaviors (RSDBs) such as gambling (Roberts et al., 2017), problematic media/technology use (Contractor, Frankfurt, et al., 2017), disordered eating behaviors (Brewerton, 2007), substance use (Clark et al., 2001), aggressive behaviors (Lusk et al., 2017), and self-injurious/suicidal acts (Spitzer et al., 2020; Stein et al., 2010). Individuals who engage in posttrauma RSDBs report functional impairment and detrimental health such as more depression severity (Contractor, Weiss, et al., 2017; Sommer et al., 2020). Furthermore, individuals who report posttrauma RSDBs (e.g., substance use) and posttrauma distress are more difficult to treat with clinical interventions and demonstrate poorer treatment outcomes/adherence (McCauley et al., 2012; Schäfer & Najavits, 2007). Indeed, clinical interventions for posttrauma symptoms have incorporated risk reduction/management techniques to mitigate RSDBs (Becker & Zayfert, 2001; Danielson et al., 2020; Senn et al., 2017).

Theoretical viewpoints help explain links between trauma and posttrauma RSDBs. For instance, the *emotion dysregulation* viewpoint suggests that trauma-exposed individuals may engage in RSDBs to reduce negative affect and increase positive affect (Ben-Zur & Zeidner, 2009; Cooper et al., 2000; Marshall-Berenz et al., 2011). Furthermore, from a *cognitive* viewpoint, trauma-exposed individuals may experience limited attention, cognitive resources, and information processing abilities, which may reduce their capacity for adaptive decisions, and thus, increase RSDBs (Ben-Zur & Zeidner, 2009). From a *disinhibition* viewpoint, trauma-exposed individuals, especially those reporting chronic childhood traumas and posttrauma psychopathology, may engage in impulsive RSDBs (Braquehais et al., 2010) when encountering situations perceived as rewarding and/or distress-reducing (Casada & Roache, 2005). Finally, the impaired risk recognition viewpoint suggests that the experience of trauma makes it difficult to accurately perceive and recognize risks in situations (Gidycz et al., 2006); such impaired judgement may contribute to RSDBs.

To comprehensively screen posttrauma RSDBs, a 16-item Posttrauma Risky Behaviors Questionnaire (PRBQ) was developed following recommended guidelines (e.g., Furr, 2011; Germain, 2006). The PRBQ examines posttrauma RSDBs covered by the *Diagnostic and Statistical Manual of Mental Disorders–Fifth edition* (*DSM-5*) E2 criterion for posttraumatic stress disorder (PTSD); examples include substance use, dangerous driving, and suicidal behavior (American Psychiatric Association, 2013). Notably, the *DSM-5* does not outline all posttrauma RSDBs intended to be captured by E2. Thus, in developing the PRBQ, the authors reviewed the literature and consulted with experts to identify additional RSDBs that are prevalent among individuals reporting trauma experiences such as disordered eating and gambling (see Contractor et al., 2020). Hereby, the PRBQ is not limiting to a symptom criterion of PTSD, which has changed across *DSM* versions; and the PRBQ can be used to examine posttrauma RSDBs spanning diverse research questions, contexts, and diagnostic constructs (not specific to PTSD). Furthermore, existing RSDB measures, such as the risky, impulsive, and self-destructive measure (Sadeh & Baskin-Sommers, 2017) or the Risky Behaviors Questionnaire (Weiss et al., 2016) could be administered with a trauma measure to examine RSDBs in the context of trauma; however, such RSDB measures were not targeted to and do not measure extent of engaging in posttrauma RSDBs (e.g., some measures examine functionality of RSDBs), and are not brief. Notably, RSDBs in the context of trauma are different in functionality, outcomes, and characteristics (beyond unique reasons linking trauma and posttrauma RSDBs as outlined earlier; Kerig, 2019). For instance, in the short-run, engaging in posttrauma RSDBs may serve to avoid, escape from, or distract from trauma-related aversive emotional/cognitive states (Cooper et al., 2000; Pat-Horenczyk et al., 2007); however, such engagement in posttrauma RSDBs may have negative consequences in the long-run (Ben-Zur & Zeidner, 2009). Furthermore, posttrauma symptoms such as arousal or dissociation, may interfere with one’s ability to avoid or escape from risky situations (Noll & Grych, 2011), increasing engagement in posttrauma RSDBs. Also, engaging in posttrauma RSDBs may help individuals reproduce the arousal linked to their trauma (Van der Kolk et al., 1985), which may explain elevated impulsivity and sensation-seeking among trauma-exposed individuals (Contractor et al., 2016; Roley et al., 2017).

Scores derived from the PRBQ have been subjected to preliminary psychometric investigations (Contractor et al., 2019; Contractor et al., 2020; Contractor et al., 2021). To-date, the PRBQ has demonstrated measurement of a unitary RSDB construct, high internal consistency (e.g., Cronbach’s α = .94), good convergent validity with distinct measures of RSDBs (including with PTSD’s E2 criterion scores assessed by the PTSD Checklist for *DSM-5* [PCL-5]), and good incremental validity (e.g., PRBQ score predicted PTSD’s E2 criterion score assessed by the PCL-5 above and beyond the Risky Behaviors Questionnaire; Contractor et al., 2020). Despite these initial strong psychometric indicators for PRBQ scores, there are a few unexplored areas. For instance, it would be helpful to examine if the existing response categories for the PRBQ items are optimal and useful (e.g., few participants in the samples used for the PRBQ validation paper endorsed “frequently and “very frequently” responses; Contractor et al., 2020); to identify groups with levels of RSDB latent trait that are most appropriate for PRBQ administration; to determine how much information each item adds to the PRBQ; and to examine differential item functioning (DIF) of PRBQ items across groups differentiated by demographic, clinical, or social characteristics.

To this end, the current study examined PRBQ’s item parameter estimates based on item response theory (IRT) which provides information on item performance in relation to an underlying latent construct assessed by a scale (Lord, 1980). IRT is an especially important tool for new measures such as the PRBQ because such IRT-based statistics help measure (a) the amount of information contributed by each item, (b) the extent to which the items distinguish individuals with versus without clinically significant levels of RSDBs, (c) the extent to which the various categories on a given scale of measurement are being endorsed for a given item, (d) whether some response patterns are misfitting the expected response pattern (i.e., whether participants respond as expected or if the PRBQ triggers unexpected response patterns), and (e) whether two individuals from two different groups who have the same level of RSDB latent trait have significantly different probabilities of endorsing a particular PRBQ item (i.e., if PRBQ items are performing differently toward a focal group relative to a reference group; Camilli & Shepard, 1994; Cole, 1993). IRT analyses of dichotomous/polytomous response data provide item- and individual-level information such as item analysis, person fit, and DIF.

First, item characteristics derived from item analysis using the graded response model (GRM) include thresholds (*b*) and discrimination (*a*) parameters. Thresholds indicate if each PRBQ item is measured by appropriate response categories; they identify items where the respondents are not choosing certain response categories or are choosing only extreme response categories. Information relating the probability of endorsing items and item parameters is portrayed figuratively with category response curves (CRCs). Furthermore, item discrimination parameters are the counterpart of slopes (steepness) in the general linear model (Baker & Kim, 2004); they indicate an item’s precision in measuring across varying RSDB latent trait levels and an item’s contribution to the RSDB latent trait. For PRBQ items with a higher discrimination parameter, the probability of choosing a response category will change more rapidly corresponding to changes in RSDB latent trait levels; these items also contribute more to the RSDB latent trait. Item and test information curves (IIC and TIC) indicate the amount of information given by each item and the overall test, respectively.

Second, through person fit estimates, we can identify atypical/aberrant response patterns indicating misfit. These person fit estimates can detect whether a person with a given RSDB latent trait level responds appropriately/atypically to a PRBQ item. This idea is also referred to as traitedness—magnitude of consistency between a person’s expected pattern of responses (i.e., indication of behavior) and theoretically anticipated response patterns on a particular dimension (Reise & Waller, 1993). Atypical responses may be attributed to psychological processes characterized by guessing, cheating, or different item interpretations (Tendeiro et al., 2016).

Finally, DIF analysis (Raju, 1988, 1990) identifies item bias, that is, whether two individuals with the same RSDB latent trait level belonging to different groups have similar or different probabilities of choosing a response category on PRBQ items. Hereby, DIF analysis can help statistically/visually determine what specific aspects of the PRBQ item are biased (i.e., items or response categories). For the current study, we first examined DIF between men and women. Indeed, research indicates that engagement in the different types of RSDBs differs across gender; men report more substance use (Brady & Randall, 1999; Nolen-Hoeksema, 2004), illegal behaviors, risky driving, and physical aggression (Pat-Horenczyk et al., 2007), while women report more dysfunctional eating (Pat-Horenczyk et al., 2007) and nonsuicidal self-injury (Bakken & Gunter, 2012). Broadly, men have been found to engage in more RSDBs (Archer, 2004; Contractor & Weiss, 2019; Grant et al., 2015). For the current study, we also examined DIF between two samples differing on some sociodemographic characteristics and method of recruitment. In this regard, engagement in RSDBs has been shown to differ across samples differing on sociodemographic and clinical characteristics: race–ethnicity defined groups differ in engagement in risky health behaviors (Daw et al., 2017); groups reporting interpersonal traumas report more RSDBs (Contractor et al., 2018); and lower socioeconomic status is associated with engagement in multiple RSDBs (Meader et al., 2016). For the current study, one sample included participants recruited from Amazon’s Mechanical Turk (MTurk) and the other sample included women recruited from the community who reported experiencing intimate partner violence (IPV) and substance use (hereby referenced as the IPV sample). The reference group, that is, the MTurk sample, had an almost uniform distribution of income, were mostly employed, and recruited online from anywhere in the United States. The focal group, that is, the IPV sample, was recruited in-person and locally from Providence County in Rhode Island, composed of only women, and had a higher percentage of individuals reporting low income and unemployment. DIF analyses would indicate if valid comparisons of RSDBs across the two different samples can be made; RSDB latent trait would be compared across the groups only if PRBQ items did not exhibit DIF across the compared groups (i.e., no bias toward a group). This is especially important given concerns of data quality for MTurk (Chmielewski & Kucker, 2020).

In summary, applying IRT and DIF, we examined and compared psychometric properties of PRBQ scores in and across two different samples. Specifically, we conducted item analysis (threshold and discrimination parameters) of PRBQ items in each sample; examined person fit estimates in each sample; and conducted DIF analysis to investigate whether the PRBQ items measured the RSDB latent trait identically across men and women (in the MTurk sample) and across the two different samples (i.e., MTurk and IPV). Broadly, stronger psychometric properties are established for an instrument that can measure the latent trait identically across different samples.

## Method

### Procedure and Participants

#### MTurk Sample

Study procedures were approved by the University of North Texas Institutional Review Board (IRB). We recruited adult participants (≥18 years) from Amazon’s MTurk, which is a crowd-sourcing platform (Paolacci et al., 2010). MTurk’s subject pool (a) is diverse compared with traditional internet-recruited samples; (b) represents the U.S. population in demographic characteristics, such as gender distribution and mean age; (c) generates reliable data (Buhrmester et al., 2011; Mischra & Carleton, 2017; Shapiro et al., 2013; U.S. Census Bureau, 2016a, 2016b); and (d) has demonstrated utility for trauma research by capturing individuals with posttrauma symptoms in a cost- and time-effective manner (Engle et al., 2020; van Stolk-Cooke et al., 2018). The current study was described as a 45- to 60-minute survey to develop a measure of posttrauma RSDBs. Eligible adult participants (a) lived in North America, (b) reported English fluency, and (c) endorsed trauma screened with the Primary Care–Posttraumatic Stress Disorder Screen for *DSM-5* (Prins et al., 2015). Eligible participants who provided informed consent and completed the Qualtrics survey validly were given $1.25.

Of the obtained 891 responses, 47 responses of 18 participants who attempted the survey multiple times were excluded (remainder *n* = 844). We further excluded 150 participants who did not meet eligibility criteria, 122 participants who did not pass all four validity checks to ensure attentive responding and comprehension (e.g., “please click on the little blue circle on the bottom of the screen;” “I am paid paid biweekly by leprechauns;” Meade & Craig, 2012; Thomas & Clifford, 2017), 97 participants who missed data on all measures, and 11 participants who did not endorse one/worst potentially traumatic event on the Life Events Checklist for *DSM-5* (LEC-5; Weathers et al., 2013). This resulted in a sample size of 464 participants. For PRBQ items, 10% of the responses were missing completely and were therefore discarded. Therefore, the final sample included 418 participants. See Table 1 for detailed demographic information.

**Table 1.** Demographic Information for the Amazon Mechanical Turk Sample (*N* = 464).

	*M* (*SD*)	*n* (%)
Age	35.7 (11.12)	
Years of education	15.23 (2.34)	
PRBQ total score	21.04 (9.78)	
Gender		
Female		258 (55.6)
Male		199 (42.89)
Male to female transgender		1 (0.21)
Female to male transgender		4 (0.86)
Other		2 (0.43)
Ethnicity		
Hispanic		60 (12.93)
Non-Hispanic		396 (85.34)
Unknown		8 (1.7)
Race		
White		355 (76.5)
African American		43 (9.2)
Asian		52 (11.2)
American Indian or Alaska Native		21 (4.5)
Native Hawaiian or Other Pacific Islander		3 (0.6)
Other, Unknown, or Multiple Races		8 (1.7)
Employment		
Full-time		325(70)
Part-time		73 (15.73)
Retired		14 (3.02)
Unemployed		41 (8.83)
Unemployed student		11 (2.37)
Income		
<$15,000		48 (10.3)
$15,000 to $24,999		59 (12.71)
$25,000 to $34,999		71 (15.3)
$35,000 to $49,999		63 (13.58)
$50,000 to $64,999		87 (18.75)
$65,000 to $79,999		43 (9.27)
≥$80,000		93 (20)
Relationship status		
Not dating		77 (16.59)
Casually dating (nonmonogamous)		34 (7.33)
Seriously dating (Monogamous)		114 (24.57)
Married		201 (43.32)
Divorced		19 (4.09)
Separated		11 (2.37)
Widowed		8 (1.72)
Potentially traumatic event (Index)		
Natural disaster		67 (14.66)
Fire or explosion		24 (5.25)
Transportation accident		74 (16.20)
Serious accident		16 (3.5)
Exposure to toxic substance		2 (0.43)
Physical assault		46 (10)
Assault with weapon		15 (3.28)
Sexual assault		58 (12.7)
Other unwanted/uncomfortable sexual experience		10 (2.19)
Combat or exposure to war-zone		5 (1.09)
Captivity		2 (0.44)
Life-threatening illness/injury		30 (6.56)
Severe human suffering		8 (1.75)
Sudden violent death		31 (6.78)
Sudden accidental death		36 (7.88)
Serious injury, harm, or death you caused to someone else		8 (1.75)
Other very stressful event/experience		25 (5.47)

*Note*. PRBQ = Posttrauma Risky Behaviors Questionnaire.

#### IPV Sample

Study procedures were approved by the University of North Texas IRB. Data were collected as part of a larger ongoing study examining day-level relations among PTSD, emotion dysregulation, substance use, and HIV/sexual risk in a sample of community women currently experiencing IPV and substance use. Participants were recruited from Providence County in Rhode Island using posters, brochures, and flyers posted in community establishments and internet forums. Inclusion criteria included (a) female gender, (b) ≥18 years; (c) English speaking; (d) involvement in a heterosexual intimate relationship with the presence of physical and/or sexual victimization; and (e) and use of drugs/alcohol (required to endorse at least one episode of alcohol use, illicit drug use, or licit drug misuse in the past month). Exclusion criteria included (a) current mania/psychosis; (b) self-reported pregnancy; (c) color-blindness; (d) cardiovascular disease; and (e) residence in a shelter/group home. Eligible participants were provided with study information, following which written informed consent was obtained. The study was completed in four parts: (a) an initial session, (b) an experimental session, (c) 30 days of thrice daily experience sampling methodology, and (d) a follow-up session. Data from the initial session were used in the current study; in this session, structured diagnostic interviews, self-report measures, and a protocol for developing emotion induction scripts were administered (Lang & Cuthbert, 1984; Lang et al., 1983). Remuneration was $40 for participation in the initial session.

The sample of 171 individuals reported a Criterion A trauma as determined by the Structured Clinical Diagnostic Interview for *DSM-5* (Brodey et al., 2016) and affirmative responses to either one of the two screening questions (“In the past 6 months, did your partner do anything to physically hurt you, such as push or shove you, grab you, or punch or hit you?” or “In the past 6 months, did your partner make you do something sexual that you didn’t want to do, such as pressuring or forcing you to do something sexual when you didn’t want to?”). See Table 2 for detailed demographic information. Data were not missing for the PRBQ items.

**Table 2.** Demographic Information for the Community Sample (*n* = 171).

	*M (SD)*	*n (%)*
Age	46.03 (74.42)	
Years of education	12.45 (1.98)	
PRBQ total score	31.27 (47.53)	
Gender		
Female		166 (97.1)
Female to male transgender		1 (0.6)
Male to female transgender		2 (1.2)
Gender queer		1 (0.6)
Other		1 (0.6)
Ethnicity		
Hispanic/Latinx		30 (17.5)
Not Hispanic/Latinx		129 (75.4)
Race		
White		70 (40.9)
African American/Black		53 (31.0)
American Indian/Alaskan Native		13 (7.6)
Hispanic/Latinx		19 (11.1)
Other		13 (7.6)
Employment Status		
Full-time		9 (5.3)
Part-time		20 (11.7)
Unemployed		107 (62.6)
Not in labor force (student, homemaker)		23 (13.5)
Annual household income		
<$15,000		67 (39.8)
$15,000 to $24,999		19 (11.2)
$25,000 to $34,999		5 (3)
$35,000 to $49,999		6 (3.5)
$50,000 to $64,999		3 (1.8)
$65,000 to $79,999		1 (0.6)
≥$80,000		4 (2.4)
Relationship status		
Married		14 (8.2)
Unmarried		127 (74.3)
Separated or divorced		13 (7.6)
Potentially traumatic event (Index)		
Natural disaster		5 (3.9)
Fire or explosion		8 (6.25)
Transportation accident		8 (6.25)
Serious accident		3 (2.34)
Exposure to toxic substance		0 (0)
Physical assault		33 (25.78)
Assault with weapon		5 (3.9)
Sexual assault		37 (28.9)
Other unwanted/uncomfortable sexual experience		4 (3.12)
Combat or exposure to war-zone		1 (0.78)
Captivity		1 (0.78)
Life-threatening illness/injury		6 (4.69)
Severe human suffering		2 (1.56)
Sudden violent death		7 (5.47)
Sudden accidental death		6 (4.69)
Serious injury, harm, or death you caused to someone else		0 (0)
Other very stressful event/experience		2 (1.56)

*Note*. PRBQ = Posttrauma Risky Behaviors Questionnaire.

### Measures

#### Life Events Checklist for *DSM-5* (Weathers et al., 2013)

Administered to both samples, the LEC-5 is a 17-item self-report measure assessing exposure to potentially traumatic experiences. Participants respond to each item with these six response options: “Happened to me,” “Witnessed it,” “Learned about it,” “Part of my job,” “Not sure,” or “Doesn’t apply.” An 18th item asked about the most potentially traumatic event. A positive response to one of the first four response options for Items 1 to16 was considered a potential trauma endorsement.

#### Structured Clinical Interview for *DSM-5* (SCID-5)

Administered to the IPV sample, a computerized version of the SCID-5 was used to ascertain trauma exposure and establish current psychiatric diagnoses (First & Williams, 2016). The SCID-5 severity scales have demonstrated good psychometric properties (Shankman et al., 2018) and there is evidence of moderate to excellent interrater reliability across major diagnostic categories (Osório et al., 2019).

#### Posttrauma Risky Behaviors Questionnaire (Contractor et al., 2020)

Administered to both samples, the PRBQ is a 16-item self-report measure of posttrauma RSDBs referencing the past month. Fourteen items measure the extent of engaging in specific posttrauma RSDBs on a response scale ranging from 0 (*never*) to 4 (*very frequently*). Two supplemental items measure functional impairment and associations between posttrauma RSDB frequency and onset of the worst trauma. The PRBQ scores have good psychometric properties (Contractor et al., 2019; Contractor et al., 2020). In the present study, ordinal Cronbach alphas were .97 and .93 for the MTurk and IPV samples, respectively; and ordinal omegas were .98 and .94 for the MTurk and IPV samples, respectively.

### Statistical Analyses

All analyses were conducted using R (R Core Team, 2019). Principal components analysis was conducted on the 14 PRBQ items using the R package *factoextra* (Kassambara & Mundt, 2020). According to Reckase (1979) and Reeve et al. (2007), the first factor should explain at least 20% of the variance in order to meet the unidimensionality assumption. Additionally, if the ratio of the first factor eigenvalue to the second factor eigenvalue <3, such an estimate also indicates unidimensionality (Morizot et al., 2007). Monotonicity in PRBQ data were examined using the R package *Mokken* (van der Ark, 2007) based on Mokken scale analysis (Mokken, 1971). The monotonicity plots were visually examined to ensure that the items were increasing monotonically. Local item dependence was tested by examining whether model fit of a single factor confirmatory factor analysis would increase by adding error correlations across items. This error correlation has to be <.20 to establish local item independence.

For IRT analyses, we used GRM (Samejima, 1968) for the PRBQ data because the item categories have a meaningful increasing order. Both constrained and unconstrained GRMs were fitted to the data using the R package *ltm* (Rizopoulos, 2006). We classified items as being low informative (LI) when the CRC peak for any response category in the PRBQ item was under another CRC throughout the entire latent trait space; this meant that not all five response category options were utilized by participants. Items were also classified as LI when the discrimination parameter of the item was <.75 (Baker, 2001). Items for which participants utilized all response categories and with discrimination parameters >.75 were categorized as being moderate to highly informative (MHI). Baker (2001) classified item discrimination parameters <.65 as moderate to low because item discrimination parameters are analogous to factor coefficients where items with higher discrimination parameters are able to better differentiate between individuals (Reiss et al., 2011). Erring on the more conservative side, we considered PRBQ items that had discrimination parameter estimates <.75 (also lower factor coefficients) and/or unutilized response categories as providing “lower” information than the other items. This being said, we acknowledge the potential arbitrary nature of the existing classifications and the lack of consensus on what defines “low” while inferring results and outlining recommendations.

Furthermore, Emons’ (2008) polytomous extension of van der Flier’s (1980, 1982) U3 statistic was computed to detect inconsistent, aberrant, or misfitting patterns of PRBQ item scores using the package *PerFit* (Tendeiro et al., 2016). Last, a DIF model (DFIT; differential functioning of items and tests; Oshima & Morris, 2008) was fit to the data to compare PRBQ item functioning across men versus women in the MTurk sample, and across the MTurk versus IPV samples using the R package *lordif* (Choi et al., 2011). Uniform DIF is indicated when the latent trait is consistently lower for one group compared to another, while the magnitude/order of the difference between the latent traits differ across groups for nonuniform DIF (Walker, 2011). The magnitude of these DIF estimates was determined using the McFadden’s *R*^2^ estimate which was classified as low when the value <10%; the difference in the group membership accounted for <10% of variation in the response patterns for the same level of the latent trait.

## Results

### Unidimensionality, Monotonicity, and Local Item Dependence

Principal components analysis revealed that the first factor explained 57.14% of the variance and the subsequent factors explained <7% of the variance. The ratio of the first factor eigenvalue to the second factor eigenvalue was 8.08. Thus, results suggested unidimensionality of the 14-item PRBQ factor. Furthermore, none of the error correlations were greater than 0.20; there was no correlation between the items after accounting for variation in the items due to variation in the factor scores. Thus, local item independence was established. Last, all the items were monotonically increasing; choosing a higher response category on a PRBQ item indicated greater engagement in posttrauma RSDBs. Therefore, the assumptions of unidimensionality, monotonicity, and local item independence for IRT analyses were met.

### MTurk Sample

#### Item Analysis

**Figure 1.**
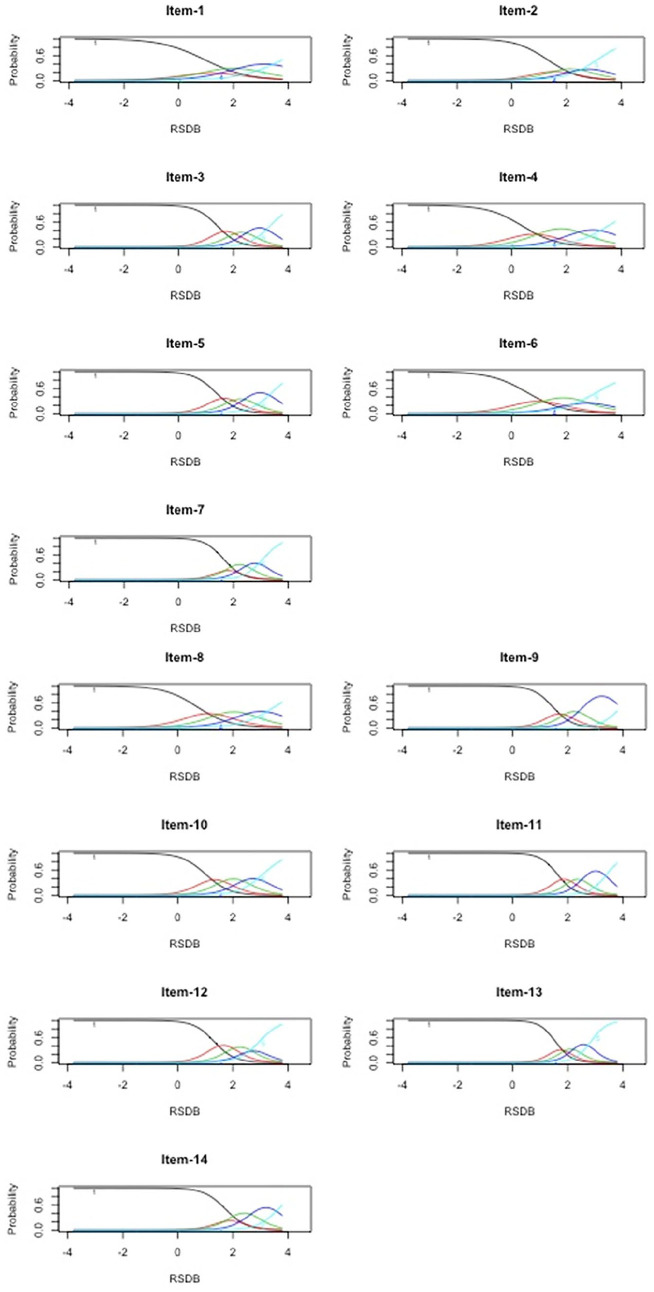
Category response curves of Posttrauma Risky Behaviors Questionnaire (PRBQ) items (MTurk Sample). *Note*. RSDB = reckless and self-destructive behaviors.

Both unconstrained and constrained GRMs had comparable fit as evidenced by similar Akaike information criterion and Bayesian information criterion values (−2[log likelihood] = 69.12, *p* < .001). Therefore, we retained the unconstrained GRM. The unconstrained GRM revealed the following decreasing order of item discriminations: 13, 11, 7, 9, 12, 3, 14, 5, 10, 2, 8, 4, 6, and 1 (see Table 3). The CRC patterns are given in Figure 1. All items had discrimination parameter values >0.75. However, all response categories were utilized for Items 3, 5, 8, 9, 10, 11, and 13 only; therefore, these items were classified as MHI. Items 1, 2, 4, 6, 7, 12, and 14 were classified as LI.

**Table 3.** PRBQ Item Parameters for the MTurk Sample and Intimate Partner Violence Sample From the Unconstrained Graded Response Model.

MTurk sample						Intimate partner violence sample				
PRBQ items	*b*_1_	*b*_2_	*b*_3_	*b*_4_	*a*	*b*_1_	*b*_2_	*b*_3_	*b*_4_	*a*
PRBQ 1	0.95	1.52	2.48	3.80	1.26	–0.44	0.76	2.07	3.41	0.81
PRBQ 2	1.31	1.81	2.48	3.13	1.73	–0.82	0.26	1.03	2.05	1.00
PRBQ 3	1.42	2.02	2.57	3.33	2.62	1.17	1.79	2.48	4.76	1.27
PRBQ 4	0.39	1.21	2.40	3.51	1.56	0.63	1.19	2.54	3.28	0.91
PRBQ 5	1.36	1.96	2.54	3.41	2.49	0.36	0.97	1.92	2.59	1.65
PRBQ 6	0.57	1.36	2.41	3.09	1.49	0.28	0.78	1.62	2.89	1.03
PRBQ 7	1.64	1.95	2.49	3.08	2.86	0.32	1.20	2.12	2.64	1.60
PRBQ 8	0.70	1.56	2.52	3.52	1.65	–0.07	0.59	1.43	2.31	1.48
PRBQ 9	1.45	1.95	2.53	3.94	2.78	0.39	1.04	1.67	2.41	2.40
PRBQ 10	1.01	1.67	2.37	3.09	2.31	–0.54	0.33	1.24	2.07	1.76
PRBQ 11	1.62	2.11	2.61	3.43	3.20	0.67	1.34	1.95	2.58	3.08
PRBQ 12	1.32	1.97	2.54	2.96	2.70	1.13	1.31	1.96	2.58	2.96
PRBQ 13	1.55	1.92	2.30	2.84	3.44	1.06	1.50	2.13	2.74	2.09
PRBQ 14	1.73	2.07	2.74	3.65	2.57	1.48	1.76	2.38	3.64	1.56

*Note*. PRBQ Item 1 refers problematic alcohol use; PRBQ Item 2 refers problematic drug use; PRBQ Item 3 refers problematic gambling; PRBQ Item 4 refers problematic technology use; PRBQ Item 5 refers impulsive/risky sexual behaviors; PRBQ Item 6 refers problematic eating behaviors; PRBQ Item 7 refers illegal behaviors; PRBQ Item 8 refers reckless spending; PRBQ Item 9 refers physically aggressive behaviors; PRBQ Item 10 refers verbally aggressive behaviors; PRBQ Item 11 refers property destruction; PRBQ Item 12 refers reckless driving; PRBQ Item 13 refers deliberately injuring self without intent to kill one self; PRBQ Item 14 refers suicidal behaviors. PRBQ = Posttrauma Risky Behaviors Questionnaire; *b* = thresholds parameter; *a* = discrimination parameter.

**Figure 2.**
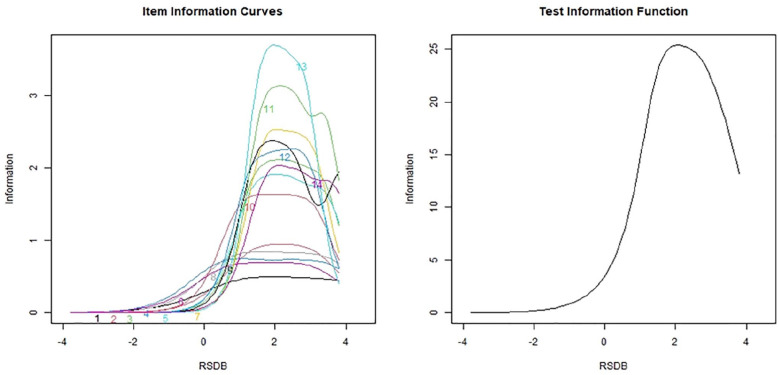
Item information curves and test information curve for the Posttrauma Risky Behaviors Questionnaire (PRBQ) items (MTurk Sample). *Note*. RSDB = reckless and self-destructive behaviors.

ICCs (see Figure 2, left panel) indicated that PRBQ Item 9 contained the least information of all the items. The information provided by all PRBQ items together was 79.73, whereas the information provided by all PRBQ items except Item 9 was 71.93 (this item contributed 9.70% of the information to the PRBQ measure). Although this is less than the information provided by the other items, this value is not negligible. Therefore, it was deemed that all the PRBQ items contributed some information to the scale. TIC (see Figure 2, right panel) indicated that >58% of the information was contained between latent trait RSDB values of 1 and 3. Additionally, 96.96% of the information was measured for latent trait level ≥0. Thus, the PRBQ provided more information on participants who endorsed a greater extent of engagement in RSDBs. In fact, all items provided maximum information between the RSDB latent trait levels of 1 and 3.

#### Person Fit Estimates

Person fit statistics (U3) revealed that eight participants did not fit the pattern of responses as expected; of these participants, three identified as male, three identified as female, one identified as male-to-female transgender, one identified as female-to-male transgender, and their ages ranged from 23 to 72 years. Variables such as relationship status, ethnicity, employment, and income levels did not explain the reason for the misfit. As an explanation for the misfit, all eight respondents endorsed all but one PRBQ item with a response category of 1; they endorsed PRBQ Items 3, 5, 7, 9, 13, and 14 with a response category of 2 or 3 (but not higher). Thus, there was no particular reason discernible from the data for these response patterns to be labelled as misfit.

#### DIF Across Gender Groups

PRBQ Items 3, 6, 8, and 13 were flagged as exhibiting DIF. The average RSDB latent trait for men and women was 1.57 and 1.40, respectively. The Cohen’s *d* standardized mean difference effect size was 0.27 which is considered small (Cohen, 1988). Thus, the men were only slightly higher on the RDSB latent trait than the women. Even when the LR χ^2^ test for nonuniform DIF [i.e., $\chi_{13}^{2}$ ) and $\mathrm{Pr}(\chi_{23}^{2})$ ] or uniform DIF [ $\mathrm{Pr}(\chi_{12}^{2})$ ] flagged items for possible DIF, McFadden’s *R*^2^ change effect size remained <8% for all the tests indicating a small effect size.

### IPV Sample

#### Item Analysis

**Figure 3.**
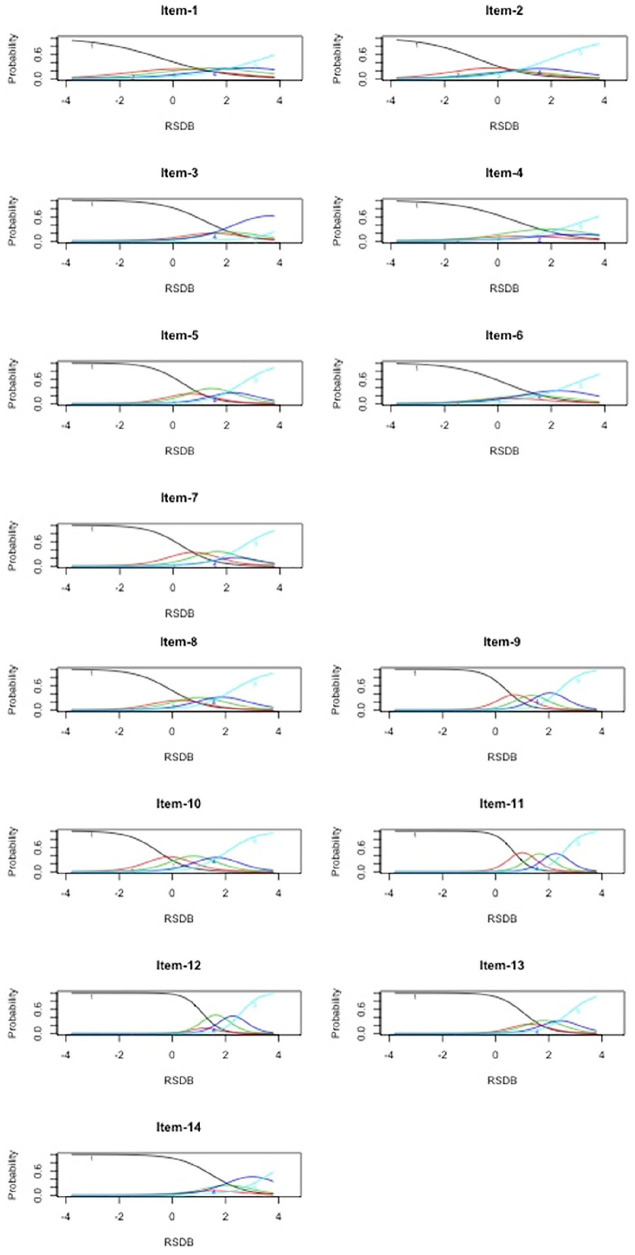
Category response curves of Posttrauma Risky Behaviors Questionnaire (PRBQ) items (intimate partner violence sample) *Note*. RSDB = reckless and self-destructive behaviors.

Both unconstrained and constrained GRMs had similar fit as evidenced by their similar Akaike information criterion and Bayesian information criterion values (−2[log likelihood] = 68.89, *p* < .001). Therefore, we retained the unconstrained GRM. The unconstrained GRM revealed the following decreasing order of item discriminations: 11, 12, 9, 13, 10, 5, 7, 14, 8, 3, 6, 2, 4, and 1 (see Table 3). The CRC patterns are given in Figure 3. Although all item discrimination parameters were > 0.75, all response categories were utilized only for Items 9, 10, and 11 (classified as MHI). The rest of the 10 items were classified as LI.

**Figure 4.**
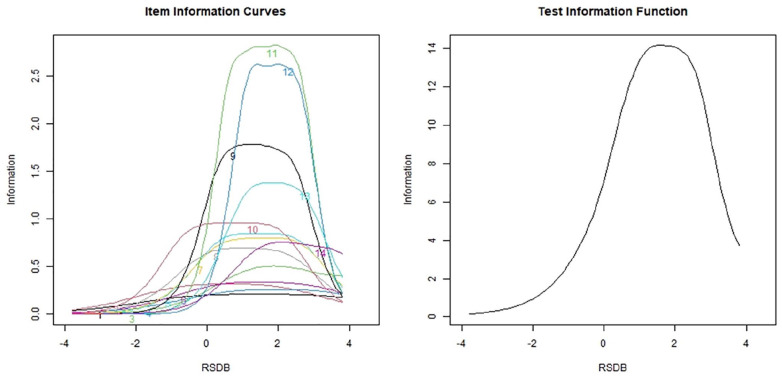
Item information curve and test information curve for the Posttrauma Risky Behaviors Questionnaire (PRBQ) items (intimate partner violence sample) *Note*. RSDB = reckless and self-destructive behaviors.

ICCs (see Figure 4, left panel) indicated that Items 1, 4, 2, and 6 contained the least information of all the items. The information provided by all items together was 53.26, whereas the information provided by all PRBQ items except these items was 46.18. Items 1 and 4 contributed 3% each, and Items 2 and 6 contributed 3.5% each indicating that each of these items contributed very little information to the entire PRBQ instrument. TIC (see Figure 4, right panel) indicated that >68% of the information was contained between latent trait RDSB values of 0.50 and 3.50. Additionally, 86.50% of the information was measured for latent trait level ≥0. This indicated that the PRBQ provided more information on participants with a greater extent of engagement in RDSBs. For this sample, all items provided maximum information for RSDB values between 1 and 3, except items such as 9 and 10 (physically and verbally aggressive behaviors), which provided maximum information at lower levels of RSDB values such as 0 to 2.5 and −1 to 2, respectively.

#### Person Fit Estimates

Person fit statistics (U3) revealed that all participants fit the pattern of responses as expected.

### DIF Analyses Across MTurk and IPV Samples

DIF analysis was conducted to compare the MTurk and IPV samples. Eight out of 14 PRBQ items were flagged for DIF (Items 1, 2, 4, 5, 7, 9, 10, and 12). The average RSDB for the IPV and MTurk samples were 1.86 and 1.49, respectively. The Cohen’s *d* standardized mean difference effect size was 0.53, which is considered medium (Cohen, 1988). McFadden’s $R^{2}$ change was <3.5% for all tests. Therefore, the amount of DIF between the groups is considered low. In sum, although the two groups differed in their RSDBs latent trait levels (IPV vs. MTurk samples exhibited more RSDB latent trait levels), there was no substantial difference in how individuals with the same amount of RSDBs belonging to the IPV or MTurk samples answered the PRBQ items.

## Discussion

The current study extends psychometric investigations of the PRBQ scores using an (IRT framework in two different samples (MTurk and IPV). Broadly, study results indicate that PRBQ items have strong psychometric properties across both samples. Considering item discrimination parameters, all PRBQ items across both samples contributed information to the understanding and measurement of the RSDB construct. In other words, all PRBQ items were able to distinguish between participants with varying RSDB latent trait levels; the probability of choosing a response category for all PRBQ items changed corresponding to changes in the RSDB latent trait levels. This being said, across samples, PRBQ Items 9 (physically aggressive behaviors), 11 (property destruction), and 13 (deliberately injuring self without intent to kill oneself) were most discriminating. Perhaps, being indicative of mental health impairment and/or poorer functioning (Armour et al., 2020; Jacobson et al., 2008; McFall et al., 1999; Selby et al., 2012) and commonly having anger regulation/expression as underlying mechanisms (Chen et al., 2012; Connor et al., 2004; Klonsky, 2007) may explain why endorsement of these items helps differentiate individuals with higher versus lower RSDB traits. Also, prior factor-analytical research has indicated relatively higher factor loadings for these items on the RSDB latent trait (Contractor et al., 2020).

Furthermore, across samples, PRBQ Items 1 (problematic alcohol use) and 4 (problematic technology use) were the least discriminating. For PRBQ Item 1, perhaps the normative U.S. culture on social drinking (Moore et al., 2005), including less stigma for alcohol use compared with other RSDBs such as drug use (because consuming alcohol does not constitute illegal behavior; Schomerus, 2014), and the high prevalence of drinking behaviors in the U.S. general population (Grant et al., 2017) may have contributed to its lesser discriminative ability across individuals endorsing higher versus lower RSDB latent trait levels. Furthermore, this single item, while a broad enough question for the purposes of PRBQ (“e.g., binge drank, which is having four or more drinks a day for women or five or more drinks a day for men; used alcohol in dangerous situations, such as driving”), could reference several facets of alcohol use for participants based on their experiences and perceptions (e.g., attempts to stop drinking, consequences/functions of alcohol use). Indeed, existing research on alcohol measures assessing these different facets has indicated differential functioning of items (Neal et al., 2006; Pilkonis et al., 2013). Referencing PRBQ Item 4, perhaps widespread use of certain technological devices (Pew Internet, 2009) for daily life purposes (e.g., work; Faulk et al., 2010) may have contributed to study findings. Also, prior factor-analytical research has indicated relatively lower factor loadings for Items 1 and 4 on the RSDB latent trait (Contractor et al., 2020).

Notably, the PRBQ abides by recommended practices of having 6/7+ response categories for optimal reliability, validity, and discriminating power (Preston & Colman, 2000). This being said, study results suggest that some PRBQ items were classified as MHI versus LI, primarily based on if all response categories were being utilized (i.e., item threshold estimates). For the MTurk sample, Items 3, 5, 8, 9, 10, 11, and 13 were classified as MHI; and for the IPV sample, Items 9 to 11 were classified as MHI. As examples, participants chose only extreme response categories for Item 2 (problematic drug use); and extreme or middle response categories for Items 1 (problematic alcohol use), 4 (problematic technology use), 6 (problematic eating behaviors), 8 (reckless spending), and 12 (reckless driving). Such results warrant an inquiry for research/clinical work into whether the currently utilized five response categories of all PRBQ items are necessary to measure extent of engagement in RSDBs; whether the response scale for certain PRBQ items may need to be revisited and modified/shortened; and whether response categories around the mid-point option are needed (Weijters et al., 2010). Notably, any reduction in response categories may facilitate responding and less participant burden/fatigue (thereby greater utility for intensive longitudinal studies); however, this will also reduce the breadth of experiences that can be reported by participants and statistically decreases variance in obtained scores (Preston & Colman, 2000). When comparing these costs of the potential loss of information vs. benefits of less participant fatigue, such response scale reduction may not be useful.

Some other points are noteworthy. While our results do not suggest deleting PRBQ items based on discrimination and threshold parameters, they imply that certain PRBQ items contributed less information for specific samples based on their characteristics. For instance, when using the PRBQ in a community sample of women currently experiencing IPV and substance use, PRBQ Items 1, 2, 4, and 6 (two of these examine substance use) contributed little information to the entire instrument. This may be a result of the substance use inclusion criteria (overlapping with PRBQ Items 1 and 2), and phone-based nature of the study (overlapping with PRBQ Item 4). Furthermore, PRBQ provides more information on participants endorsing greater extent of engagement in RDSBs. Thus, maximum information could be obtained from the PRBQ for participants with higher levels of the RSDB latent trait across both samples. Regarding person fit estimates, participants chose responses as expected for their RSDB latent trait levels and there was no evidence that the PRBQ kindled any adverse psychological reactions for participants. Last, in terms of DIF results, PRBQ items functioned well across gender-based groups as well as across different samples. Although several items were flagged for potential DIF, their effect sizes were low indicating that there was no difference in how two individuals from different groups (gender-based groups or samples) responded to the PRBQ items if they had the same RSDB latent trait level.

Current study results need to be interpreted in the light of limitations. First, study findings are limited by the demographic characteristics of the samples under consideration; future research needs to examine the study’s research questions in other racially/ethnically and clinically diverse samples. Second, we used gender as a dichotomous variable primarily for sample size considerations; however, this approach did not capture functioning of the PRBQ across all gender-based groups. Third, future investigations may benefit from examining IRT-related psychometric properties in longitudinal investigations; such longitudinal item invariance to ensure that the RSDB construct can be compared across time is especially important when investigating intervention impacts. Fourth, GRM usually requires sample sizes of at least 200 (Kieftenbeld & Natesan, 2012). In this regard, the IPV sample size was lower than this recommended sample size; however, the MTurk sample met these guidelines. Fifth, the item discrimination parameter value cut-off estimate as greater than 0.75 is arbitrary. However, given that all discrimination parameters were much higher than 0.75, we believe this limitation is of less consequence to the implications of the current study findings.

Sixth, the self-report LEC-5 used in the current study, especially in the context of the MTurk sample, is a screener for potentially traumatic events. For the MTurk sample, we did not use an interview format of a trauma measure; hence, cannot ascertain if individuals endorsed a Criterion A trauma as indicated in the *DSM-5*. Also, the LEC-5 has limited psychometric investigations and there are concerns about individuals not reporting potentially traumatic events on the LEC-5 reliably across time (Pugach et al., 2021). Last, recruitment via the MTurk platform has some concerns: the sample may include self-selected participants (Kraut et al., 2004); there is lack of control over the research environment (Thomas & Clifford, 2017); obtained data may have low quality (Chmielewski & Kucker, 2020); individuals may attempt the survey multiple times or fake study eligibility (Hauser et al., 2019); and computer programs may automatically complete research studies (Chmielewski & Kucker, 2020). While we followed recommended steps to enhance data quality such as use of validity checks to ensure attentive responding and comprehension, and excluded individuals missing too much data or attempting the survey multiple times (Aust et al., 2013; Buhrmester et al., 2011; Hauser et al., 2019; Oppenheimer et al., 2009), such steps also contributed to sample truncation (~52%). This potential selection bias in our study may limit generalizability of study results. Future research could use other quality checks such as restricting participation to MTurk workers with high reputation (>95% approval ratings; Peer et al., 2014).

Despite these limitations, current study results have important implications. Broadly, study results indicate all PRBQ items contributed information to the understanding and measurement of the RSDB construct and that the PRBQ items functioned well psychometrically across the MTurk and the IPV samples, individually. Importantly, these findings indicate that the PRBQ can be administered meaningfully across populations with different socio-demographic characteristics and recruitment modality (online vs. in-person). Furthermore, the PRBQ items exhibited negligible DIF across gender-based groups or across the different samples. That is, given the same amount of the RSDB latent trait, the probability of endorsing any item on any response category was identical across men and women as well as across the MTurk and IPV samples. Thus, the results provide support for clinicians and researchers to compare PRBQ-assessed RSDBs across these groups. Last, all PRBQ items provided maximum information on participants with a greater extent of engagement (latent trait RSDB levels between 1 and 3). Therefore, it might be useful to include more items that can provide further information on participants reporting less engagement in RSDBs (i.e., lower values of RSDB latent trait). Broadly, results support strong psychometric properties of the PRBQ scores and PRBQ’s use for clinicians and researchers.
